# Editorial: Space and neural cell: the impact of space environment on neurological function and their molecular mechanistic insights

**DOI:** 10.3389/fncel.2024.1454014

**Published:** 2024-07-12

**Authors:** Zixuan Chen, Zongjian Liu, Di Wu, Yulin Deng

**Affiliations:** ^1^Beijing Laboratory for Separation and Analysis in Biomedicine and Pharmaceuticals, School of Medical Technology, Beijing Institute of Technology, Beijing, China; ^2^Beijing Rehabilitation Hospital, Capital Medical University, Beijing, China; ^3^Department of Neurology and China-America Institute of Neuroscience, Xuanwu Hospital, Beijing Institute of Brain Disorders, Capital Medical University, Beijing, China

**Keywords:** space environment, central nervous system, microgravity, radiation, cognition impairment, immune activation

As manned space explorations continues to grow in many nations, it is crucial to research how the space environment affects astronaut health in order to get ready for upcoming, extended lunar and Mars landing missions (Garrett-Bakelman et al., 2019; da Silveira et al., 2020). Research into the viability of long-duration space flight began in the late 1950s and was carried out extensively by both the US and the Soviet Union, progressively increasing mission lengths from days to months or even years (Wei et al., 2018). A successful space mission counts not only on the advanced technology and precise instruments aboard the spaceship but also on the astronauts' ongoing wellbeing and productivity.

The space environment, extensively varied from the unique environment of Earth, is inundated with many extraterrestrial conditions such as microgravity, high-energy radiation, irregular sleep cycles, noise pollution, and loneliness (Garrett-Bakelman et al., 2019). The central nervous system (CNS), a vital central pivot in astronauts' operational performance, is more susceptible to these conditions, affecting their mental health, motor coordination, and cognitive abilities (Roy-O'Reilly et al., 2021; Marfia et al., 2022). Therefore, this Research Topic encompasses the concerns encountered by astronauts in space by examining and elucidating the molecular mechanisms behind the immediate and long-term impacts of space travel on the CNS. Moreover, these in-depth studies offer meritorious insights and opportunities for addressing scientific challenges in space and on Earth. Our aim is to accelerate the field of space neurobiology by studying the neurological alterations brought about by space missions, with the goal of improving human health and quality of life.

An increasing amount of research has investigated the intricate interactions involved in the immune system and the brain (Salvador et al., 2021). Microglia, acting as the key sentinels, are essential for guarding overall brain health and assisting in its recovery from pathological disruptions. They are active responders in the polytropic physiological and pathological conversations within the brain, changing their functions by subtly interacting with neural networks (Li et al., 2023). However, these interactive activities may undergo prominent changes under the paradigm of a space environment. The space environment, specifically microgravity, may influence the functions and behaviors of immune cells to impact the nervous system. Similarly, an understanding of the brain's immune cell functions remains vital in the contexts of Earth and space for maintaining neurological health and the formulation of efficacious strategic measures (Rienecker et al., 2021). A review article by Li et al. demonstrated the potential risks of microglial activation and their role in spaceflight-induced cognitive dysfunction and neurological health. Their comprehensive analysis of the neurological hazards induced by microglial activation revealed that stress factors may trigger microglial activation in the space environment, including Galactic Cosmic Rays (GCRs), microgravity, isolation, and stress exposure. Thus, it emphasizes that microgravity is a major concern during spaceflight and triggers microglia activation that results in nervous system damage, consequently impacting the neurologic health of astronauts (Li et al.).

The hippocampus is known to play a significant role in learning, memory, and spatial awareness within the brain. Interestingly, its adaptive responses under microgravity are significant for understanding the intricate regulatory mechanisms of cognition, thereby suggesting a foundational framework for devising cognitive safeguarding strategies within the realm of space exploration. Ming and his team revealed the adaptive effects on hippocampal transcriptome and synaptic function and their impact on cognitive function under the simulated microgravity. The findings revealed a complicated interaction in the context of simulated microgravity, which improved the animals' working memory and cognitive abilities but at the expense of impaired synaptic plasticity in the crucial CA3–CA1 hippocampal circuitry. Using RNA sequencing, the group found significant changes in the hippocampal transcriptome after acute and long-term simulations of microgravity. These results suggest that time-dependent regulation of hippocampal gene expression and dynamic changes in synaptic function may be the underlying mechanisms behind the maintenance or augmentation of cognitive performance (Liang et al.). Moreover, comparable research has demonstrated the fundamental mechanism of action of the microgravity environment and validated its significant impact on cognitive function. For instance, a 42-day simulated long-term spaceflight experiment in rats revealed that compound stress reduced the expression of important NMDA receptor channel subtypes, NR2A and NR2B, and caused weight loss, anxiety, and depression in rats. It also inhibited synaptic plasticity by impairing synaptic structure and function in the hippocampal CA1 region, particularly long-term potentiation (LTP) (Yin et al., 2024). Another study discovered that mice showed signs of neuronal senescence and cognitive decline in the hippocampus upon microgravity simulation that influenced the IGF2BP2-Shox2 axis, which controls neuronal aging and cognitive function, by altering the m6A gene. They thus postulate that IGF2BP2 and Shox2 overexpression might serve as potential treatment targets for preserving cognitive function during space travel to mitigate the neurodegenerative consequences and cognitive impairment due to microgravity conditions (Zhao et al., 2024).

In addition to microgravity, space radiation-GCRs and Solar Particle Events (SPEs)-also significantly jeopardizes astronauts' wellbeing. And certain types of radiation have both acute and long-term impact on the CNS of astronauts, potentially bringing about altered behavior, weakened motor function, and cognitive dysfunction. Premature aging and neurological disorders like Alzheimer's disease (AD) may also become more likely because of this extended exposure. While the neurocognitive and behavioral consequences were shown in radiation-exposed animal models, and harmful CNS changes have been seen in cancer patients receiving high-dose radiation therapy on Earth, the precise implications of these findings for astronauts are yet unknown (Nelson et al., 2016). Recently, Ma's research team found persistent neuroinflammation and neuronal damage in the rat striatum after exposure to heavy ions of different doses (15Gy of ^12^C^6+^ and both 3.4Gy and 8Gy of ^56^Fe^26+^) and durations (1, 2, and 3 months), respectively. And these adverse effects are worsened by the eccentric activation of the immune system. Furthermore, the visible effects of heavy ions on different kind of immune cells were also corroborated, presenting the suppression of monocyte migration and differentiation and the encouragement of monocyte survival. These findings underscore the need for thoroughly understanding the crosstalk within immunological and neural cells triggered by heavy ions (Chen et al.).

This Research Topic of the journal aims to build a bridge for scientific researchers in this field to broaden their perspective of the pathological alterations in the CNS brought about by the space environment and how these alterations play vital roles in the deterioration of cognitive abilities, the amplification of neuroinflammatory reactions, and the emergence of neurodegenerative illnesses. Our aim is to enhance the understanding of the vulnerabilities of the CNS in space and offer a rationale for scientific research into the creation of mitigation techniques for potential space hazards borne by astronauts during space missions.

## Author contributions

ZC: Writing – original draft, Writing – review & editing. ZL: Writing – review & editing. DW: Writing – review & editing. YD: Supervision, Writing – review & editing.
